# M^6^A-Mediated Upregulation of LINC00106 Promotes Stemness and Metastasis Properties of Hepatocellular Carcinoma via Sponging Let7f

**DOI:** 10.3389/fcell.2021.781867

**Published:** 2021-11-11

**Authors:** Wenjin Liang, Yan Wang, Qinyu Zhang, Min Gao, Haizhou Zhou, Zhenran Wang

**Affiliations:** ^1^ General Surgery, Affiliated Hospital of Guilin Medical University, Guilin, China; ^2^ Hubei Key Laboratory of Medical Technology on Transplantation, Transplant Center of Wuhan University, Zhongnan Hospital of Wuhan University, Institute of Hepatobiliary Diseases of Wuhan University, Wuhan, China

**Keywords:** Hepatocellular carcinoma, LINC00106, Let7f, stemness, metastasis, periostin

## Abstract

**Background:** Hepatocellular carcinoma (HCC) cells exhibit the stemness property, which makes the patient with HCC prone to tumor recurrence and metastasis. Despite the prominent regulatory role of long non-coding RNAs (lncRNAs) in tumor stemness, the roles and molecular mechanisms of LINC00106 in HCC are poorly understood.

**Methods:** LINC00106, let7f and periostin expression levels in tissue specimens and cell lines were assessed through qRT-PCR and immunohistochemistry (IHC). Various *in vivo* and *in vitro* assays, namely sphere/colony formation, proportion of side population cells (SP%), invasion, migration, western blot, and murine xenograft model were employed for assessing the stemness and metastatic properties of HCC cells. Luciferase reporter assays, RNA-seq, RNA pull-down, RNA immunoprecipitation (RIP) were conducted to clarificate the target gene and analyze the underlying mechanisms.

**Results:** LINC00106 was prominently upregulated in tissues and cell lines of HCC. Patients having a high LINC00106 level exhibited a poor outcome. Under *in vivo* and *in vitro* conditions, the stemness and metastatic properties of HCC cells were augmented by LINC00106. Additionally, LINC00106 was found to sponge let7f to upregulate periostin, which lead to the activation of periostin-associated PI3K-AKT signaling pathway. Moreover, m^6^A methylation was found to cause LINC00106 upregulation while maintaining LINC00106 RNA transcript stability.

**Conclusion:** m^6^A methylation triggers the upregulation of LINC00106, which promotes the stemness and metastasis properties in HCC cells by sponging let7f, thereby resulting in periostin activation. The findings indicate the potential of LINC00106 as a diagnostic marker and therapeutic target for HCC.

## Introduction

Hepatocellular carcinoma (HCC) is the fourth most common and lethal malignancy globally (Kelley and Greten, 2021). Several studies have confirmed the presence of a distinct tumor cell subpopulation with stemness property, known as the cancer stem cell (CSC) population, in HCC (Ling et al., 2019; Ma et al., 2020). The CSC population possesses the stemness property, which can make the patients prone to tumor invasion and metastasis (Chen et al., 2021), accounting for the extremely poor prognosis of most patients at the time of HCC diagnosis (Faltas, 2012). Therefore, the specific reprogramming mechanisms of CSCs in HCC must be studied to develop an optimal treatment strategy for improving the prognosis of patients with HCC.

Long non-coding (lnc) RNAs represent non-protein-coding RNAs with a length exceeding 200 nucleotides. These RNAs are involved in the acquisition of all carcinoma hallmarks, such as inherent proliferation and survival capabilities through metabolic enhancement and linkage to the carcinoma micro-environment (Statello et al., 2020). Additionally, in several carcinomas, lncRNAs have been proven to critically regulate the stemness and metastatic properties (Zheng et al., 2019; Shu et al., 2021). Mechanical research has demonstrated that the lncRNAs inhibit the miRNA-mediated inhibition of downstream target gene expression by acting as miRNA sponging molecules (cytosolic lncRNAs) (Zuo et al., 2020), which otherwise functionally support the regulatory protein recruitment to corresponding target chromosomal zones (Yuan et al., 2017). As a newly identified molecule of lncRNA, the effects of LINC00106 on carcinoma progression remain mostly unclarified.

In this study, we demonstrated that LINC00106 was significantly upregulated both in HCC tissue and cell lines. Patients with a high level of LINC00106 had a poor prognosis. LINC00106 could enhance the stemness and metastasis properties *in vitro* and *in vivo* in HCC cells. Moreover, LINC00106 could sponge let7f, which caused periostin upregulation and the activation of downstream signaling molecules such as p-AKT and p-PI3K. Moreover, m^6^A methylation was found to cause LINC00106 upregulation while maintaining LINC00106 RNA transcript stability. The results of this study emphasize that LINC00106 can facilitate metastasis in HCC through let7f sponging and periostin upregulation. This study indicated a potential role of LINC00106 as a novel diagnostic and treatment target for HCC.

## Materials and Methods

### Patient Samples

This study was approved by the Ethics Committee of the Affiliated Hospital of Guilin Medical University, and all patients signed an informed consent formulated under the Declaration of Helsinki principles. A total of 171 patients with HCC, aged between 30 and 70 years, who underwent hepatectomy from 2014 to 2020 were enrolled. The clinical and pathological data included sex, age, tumor diameter, tumor differentiation, and clinical TNM staging. All the enrolled patients were followed up.

### Cell Lines and Cell Culture

HCC cell lines (MHCC-97H, SNU-449, Huh7, Hep3B, BEL-7405, and HCC-LM3) and normal liver cells (THLE-2) were purchased from the Cell Bank of Chinese Academy of Sciences (shanghai, China). DMEM (Thermo Fisher Scientific, South America) was used as a culture medium for Huh7, HCC-LM3, Hep3B, and MHCC-97H cells, whereas RPMI-1640 from the same company was used for BEL-7405, SNU-449 and THLE-2 culture. These media contained 10% FBS from the same company. Cells were cultured in a 37°C incubator under 5% CO_2_/95% atmosphere. Unless otherwise stated, all the chemicals and relevant reagents were purchased from Sigma-Aldrich (Saint Louis, MO, United States).

### Vector Constructs, Lentivirus Production, and Cell Transduction

LINC00106-shRNA plus negative control, LINC00106-overexpressing vector pcDNA3.1 (+) plus corresponding control, and the mimics and inhibitor of let7f plus their negative controls were obtained from GenePharma (Shanghai, China). Mettl3 and IGF2BP1 Short interfering (si) RNA sequences were synthesized directly at GenePharma (Shanghai, China), whereas human periostin sequences (full-length) were cloned into the pcDNA3.1 vector in the presence or absence of a Flag- or HA-tag sequence (Invitrogen, Shanghai, China). Cellular transfection of shRNA and pcDNA3.1 was performed using Lipofectamine 3000 (Invitrogen, Shanghai, China), and after 48 h, the cells were collected for experimental use. For delivery of shRNAs, infection with lentiviruses generated from pLKO.1 vector-based transfection of 293T cells was performed. The lentivirus-infected cells delivering scrambled shRNA were adopted as negative control. Supplementary Table S1 provides the sequences of the shRNA, siRNA, and mimics.

### Detection of HCC Stem Cell Characteristics

After seeding HCC cells into the ultralow attachment microplates at a density of 3,000 cells per well, the cells were cultured for 10 days in DMEM/F12 medium (Invitrogen, Shanghai, China) containing insulin (4 mg/ml; Sigma, Shanghai, China), EGF (20 ng/ml; Sigma, Shanghai, China), bFGF (20 ng/ml; Sigma, Shanghai, China), and B27 (1:50, GIBCO, Shanghai, China). This procedure was followed by the acquisition of primary spheres, trypsin-based dissociation of cells, and replating, which resulted in the generation of secondary spheres through the same procedure. Finally, the sphere number was counted using a microscope (Zeiss, Germany), and the proportion of side population cells was counted by flow cytometry (Merck Millipore). Results are presented as means ± SD of triplicate wells for each experiment.

### RNA Immunoprecipitation

We used the Magna RIP RNA-Binding Protein Immunoprecipitation Kit (Millipore, United States) according to the manufacturer’s instructions to perform RIP experiments. Briefly, cell extracts were immunoprecipitated with sepharose beads conjugated antibodies against AGO2, IGF2BP1, or IgG at 4°C for 6 h. 0.1% 10 SDS/Proteinase K (0.5 mg/ml, 30 min at 55°C) was used to remove proteins from the complex. Then, Western blot and qRT-PCR were used respectively to detect the immunoprecipitated proteins and RNAs. For MeRIP-qPCR assay, m^6^A-modified RNA was eluted twice with 6.7 mM N6-methyladenosine 5′-monophosphate sodium salt at 4°C for 1 h. Subsequently, qRT-PCR analysis was performed to determine the m^6^A enrichment on LNC00106 using the following primer sequences (Supplementary Table S1).

### RNA Pull-Down Assay

Pierce™ Magnetic RNA-Protein Pull-Down Kit (Thermo Fisher Scientific, 20164) was used according to the manufacturer’s instructions. Briefly, LINC00106 was labeled for attachment to streptavidin magnetic beads, which can capture protein complex combined with labeled LINC00106. LINC00106 and let7f were detected by qRT-PCR and AGO2 was assessed by western blot.

Immunohistochemistry (IHC), Western blot analysis, Quantitative real time polymerase chain reaction (qRT-PCR), Plate cloning experiment, Experiments of cell invasion and migration, *in vivo* tumor growth assay and nude murine xenograft model, Dual luciferase reporter assay, RNA-seq analysis. Details are provided in the Supplementary Material (Additional file 2).

### Statistical Analysis

Statistical analyses were performed using GraphPad Prism 5 (GraphPad Software, Inc., San Diego, CA, United States) and SPSS v.18.0 (SPSS Inc., Chicago, IL, United States). All data are presented as mean ± SD. Pairwise comparisons were performed based on the two-tailed Student’s t-test. One-way ANOVA was performed to compare differences between at least three groups. The relationships between the expression of LINC00106 and the clinicopathologic features were assessed using the chi-square test. The Kaplan-Meier method was used to plot survival curves, which were compared using the log-rank test. A *p* value of <0.05 was considered statistically significant.

## Results

### LINC00106 is Upregulated in HCC and is Positively Correlated With Poor Prognosis in Patients With HCC

**FIGURE 1:**
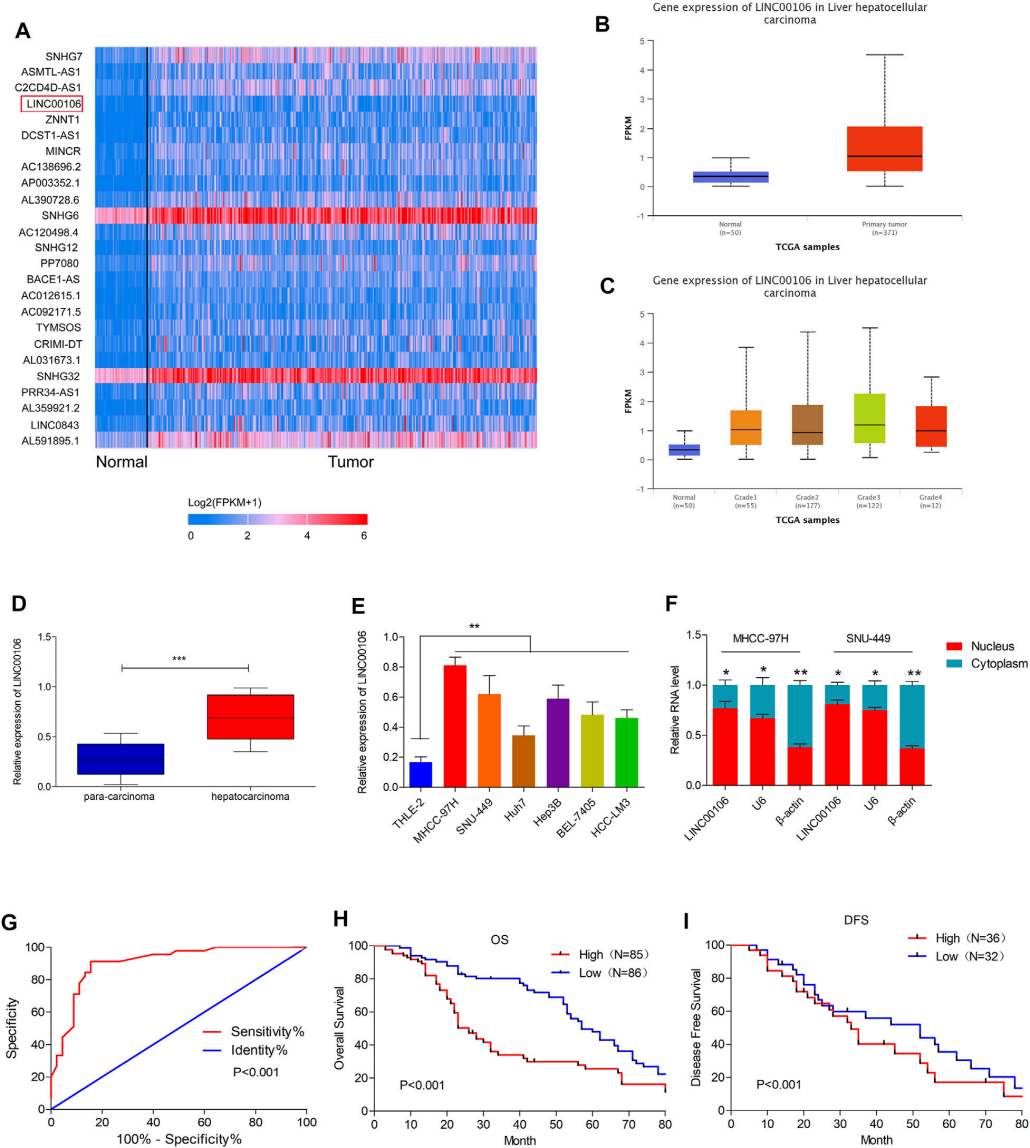
LINC00106 is up-expressed in HCC and is positively associated with poor prognosis in HCC patients. **(A)**, The heatmap of the differentially expressed LncRNAs. Between adjacent normal liver tissues (50 cases) and HCC tissues (371 cases) across TCGA database. **(B,C)**, Comparison of LINC00106 expression between 371 HCC tissues and 50 adjacent normal liver tissues based on TCGA dataset. **(D)**, Expression of LINC00106 between 171 pairs of HCC tumor tissues and adjacent normal liver tissues. **(E)**, Relative expression of LINC00106 in HCC cell lines and THLE-2 cells. **(F)**, The expression of LINC00106 in the nucleus and the cytoplasm in HCC cell lines. **(G)**, The receiver operator characteristic (ROC) analysis determining the diagnostic value of LINC00106 expression level in differentiating between normal and malignant hepatic tissues. **(H,I)**. Kaplan-Meier analyses for the correlation between the LINC00106 level and HCC prognosis in 171 patients **(H)**. Overall survival; **(I)**. Disease free survival) (*p < 0.05, **p < 0.01).

Initially, upregulated lncRNAs were screened in the HCC tissues (371 cases) and non-cancerous hepatic tissues (50 cases) through the TCGA database (Figure 1A). In HCC tissues, we observed a considerably high expression of LINC00106, whose level was positively associated with the tumor differentiation grade of HCC (Figures 1B,C). To confirm these results, we selected HCC and paracarcinoma tissues (82 pairs) for LINC00106 level measurement through qRT-PCR. The result demonstrated a high expression of LINC00106 in HCC tissues (Figure 1D). Additionally, LINC00106 was found to be significantly upregulated in HCC cell lines (MHCC-97H, SNU-449, Huh7, Hep3B, BEL-7405, and HCC-LM3) compared with that in the non-cancerous hepatic THLE-2 cell (Figure 1E). We also found that, in HCC cell lines, the expression of LINC00106 in the nucleus is higher than that in the cytoplasm (Figure 1F).

Furthermore, we explored the correlation between upregulated LINC00106 and the clinicopathological characteristics of patients with HCC. The receiver operator characteristic (ROC) analysis demonstrated that upregulated LINC00106 had a remarkable diagnostic value in discriminating between HCC and adjacent healthy tissues (AUC = 0.905, *p* < 0.001) (Figure 1G). Furthermore, the Kaplan–Meier survival analysis indicated that the patients with a high LINC00106 level exhibited a poor prognosis both in overall survival (Figure 1H) and disease-free survival (Figure 1I). In addition, the upregulated LINC00106 was positively correlated with tumor diameter (*p* < 0.05) and TNM stage (*p* < 0.05; Table 1). These results suggested that LINC00106 may have an oncogenic role in HCC.

**TABLE 1:** Correlations between LINC00106 expression and clinicopathological parameters in 171 hepatocellular carcinoma patients.

Variables	LINC00106 expression		Total	*p* Value
	Low (54)	High (117)		
Age (y)				
<60	20 (26%)	56 (74%)	76	0.188
≥60	34 (45%)	61 (55%)	95	
Gender				
Male	31 (34%)	61 (56%)	92	0.524
Female	23 (29%)	56 (71%)	79	
Tumor diameter (cm)				
<5	12 (13%)	79 (87%)	91	**0.001**
≥5	42 (52%)	38 (48%)	80	
AFP (ng/ml)				
<20	21 (33%)	42 (67%)	63	0.708
≥20	33 (32%)	75 (68%)	108	
Tumor differentiation				
Well	13 (27%)	36 (73%)	49	0.585
Moderate	19 (37%)	32 (63%)	51	
Poor	22 (31%)	49 (69%)	71	
TNM stage				
I–II	19 (22%)	68 (88%)	87	**0.005**
III–IV	35 (41%)	49 (59%)	84	
Alcohol history				
No	28 (22%)	72 (88%)	100	0.235
Yes	26 (41%)	45 (59%)	71	

Bold values represents Significant p value.

### LINC00106 Promotes HCC Cell Stemness and Metastasis Properties *in vitro*

**FIGURE 2:**
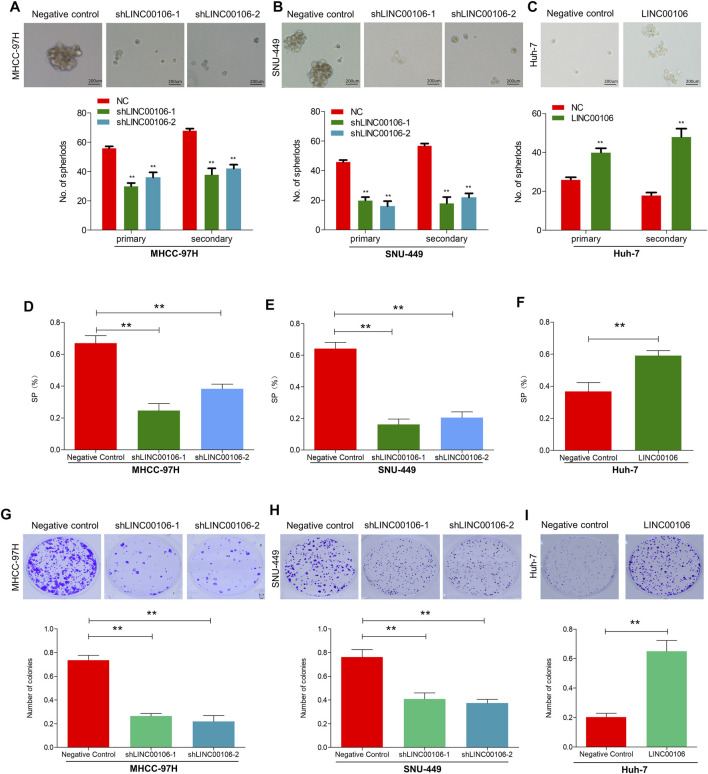
LINC00106 promotes HCC cell stemness and metastasis properties *in vitro*. **(A–C)**, Representative images of sphere formation induced by the transfection of shLINC00106 into MHCC-97H and SNU-449 cells or the transfection of a LINC00106 overexpression plasmid into Huh7 cells. **(D–F)**, SP% was detected by the above HCC cells. **(G–I)**, The plate cloning experiment was detected by the above HCC cells (**p < 0.01).

To explore whether LINC00106 can promote the stemness and metastasis properties of HCC tumors, we selected two HCC cell lines (MHCC-97H and SNU-449) with a high expression level of LINC00106 and a HCC cell line (Huh7) with a relatively low expression level of LINC00106. We downregulated the expression of LINC00106 in MHCC-97H and SNU-449 cell lines and upregulated its expression in the Huh7 cell line. By using the spheroid formation assays and side population cell screening experiment, we attempted to verify that LINC00106 regulates the characteristics of tumor stem cells of HCC cells. The sphere (primary and secondary) formation capabilities were weakened by abnormal LINC00106 suppression in MHCC-97H and SNU-449 cells compared with that in the control cells (Figures 2A,B). By contrast, in Huh7 cells, the sphere (primary and secondary) formation capabilities were enhanced due to LINC00106 overexpression (Figure 2C). The cell ratio indicated that the ectopic suppression of LINC00106 reduced SP% in the MHCC-97H and SNU-449 cells compared with that in the control cells (Figures 2D,E), whereas LINC00106 overexpression enhanced SP% of Huh7 cells (Figure 2F).

**FIGURE 3:**
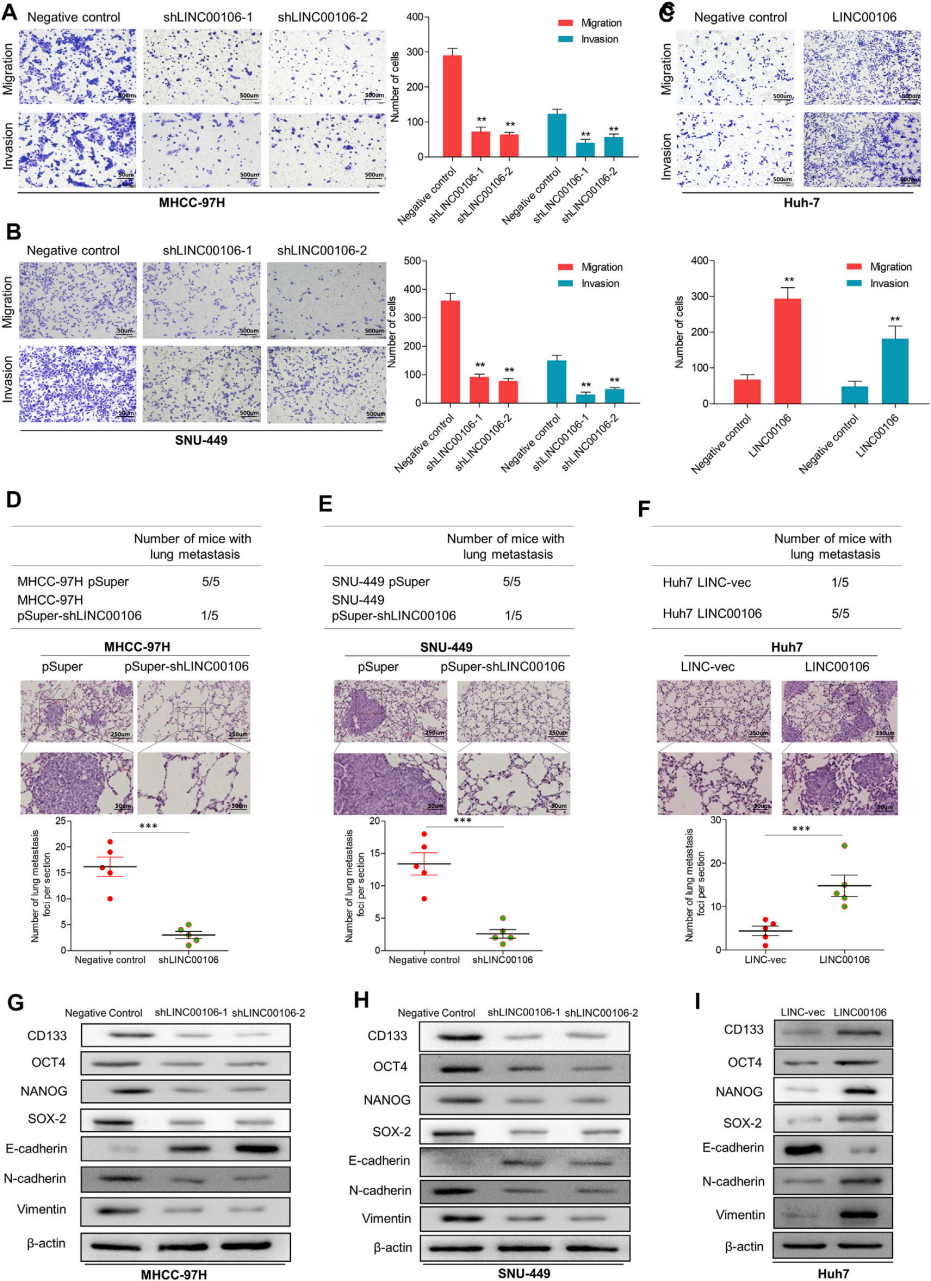
LINC00106 promotes HCC cell stemness and metastasis properties *in vitro* and *in vivo*. **(A–C)**, Representative images of invasion and migration assays induced by the transfection of shLINC00106 into MHCC-97H and SNU-449 cells or the transfection of a LINC00106 overexpression plasmid into Huh7 cells. **(D–F)**, the foci number of lung metastases were detected by the above HCC cells. **(G–I)**: the expression levels of stemness and metastasis properties related proteins were detected in the above three groups of subcutaneous tumour (*p < 0.05, **p < 0.01).

Additionally, we verified through plate cloning experiments that the ectopic suppression of LINC00106 can attenuate the colon formation ability of MHCC-97H and SNU-449 cells compared with that of the control cells (Figures 2G,H), whereas LINC00106 overexpression enhanced the colon formation ability of the Huh7 cells (Figure 2I). According to the transwell chamber invasion and migration experiments, the invasion and migration abilities of MHCC-97H and SNU-449 cells clearly decreased after downregulation of LINC00106 (Figures 3A,B), whereas those in Huh7 cells were enhanced after LINC00106 was upregulated (Figure 3C). These results suggested that LINC00106 has an oncogenic role in promoting stemness and metastasis properties in HCC cells.

### LINC00106 Promotes the Metastasis Properties of HCC Cells *in vivo*

To verify the effect of LINC00106 on the metastasis properties of HCC cells *in vivo*, LINC00106 was downregulated in MHCC-97H and SNU-449 cells and upregulated in Huh7 cells; these cells were injected via subcutaneous inoculation of cells into nude mice. The mice were sacrificed through cervical dislocation after 60 days. The tumor growth decreased significantly after LINC00106 downregulation (Supplementary Figures S2A,B,D,E). While the tumor growth increased significantly after LINC00106 upregulation (Supplementary Figures S2C,F). Besides, the above cells were also injected into the tail vein of nude mice. Then, the pulmonary metastatic foci were recorded. The number of foci of lung metastases decreased significantly after LINC00106 downregulation (Figures 3D,E). Conversely, the number of foci of lung metastases increased significantly after LINC00106 upregulation (Figure 3F). Furthermore, we collected subcutaneous tumor from different groups of nude mice and determined the expression levels of proteins related to stemness and metastasis. According to western blot results shown in Figures 3G,H, after LINC00106 was silenced, the markers related to stemness and metastasis, such as CD133, NANOG, N-cadherin, SOX-2, OCT4, and vimentin, exhibited a prominent downregulation. By contrast, LINC00106 overexpression promoted significant upregulation of stemness- and metastasis-associated markers (Figure 3I). These results confirmed that LINC00106 promotes the metastasis properties of HCC cells *in vivo*.

### LINC00106 can Sponge and Decrease the Expression of MiR-let7f in HCC Cells Through CeRNA Mechanism

**FIGURE 4:**
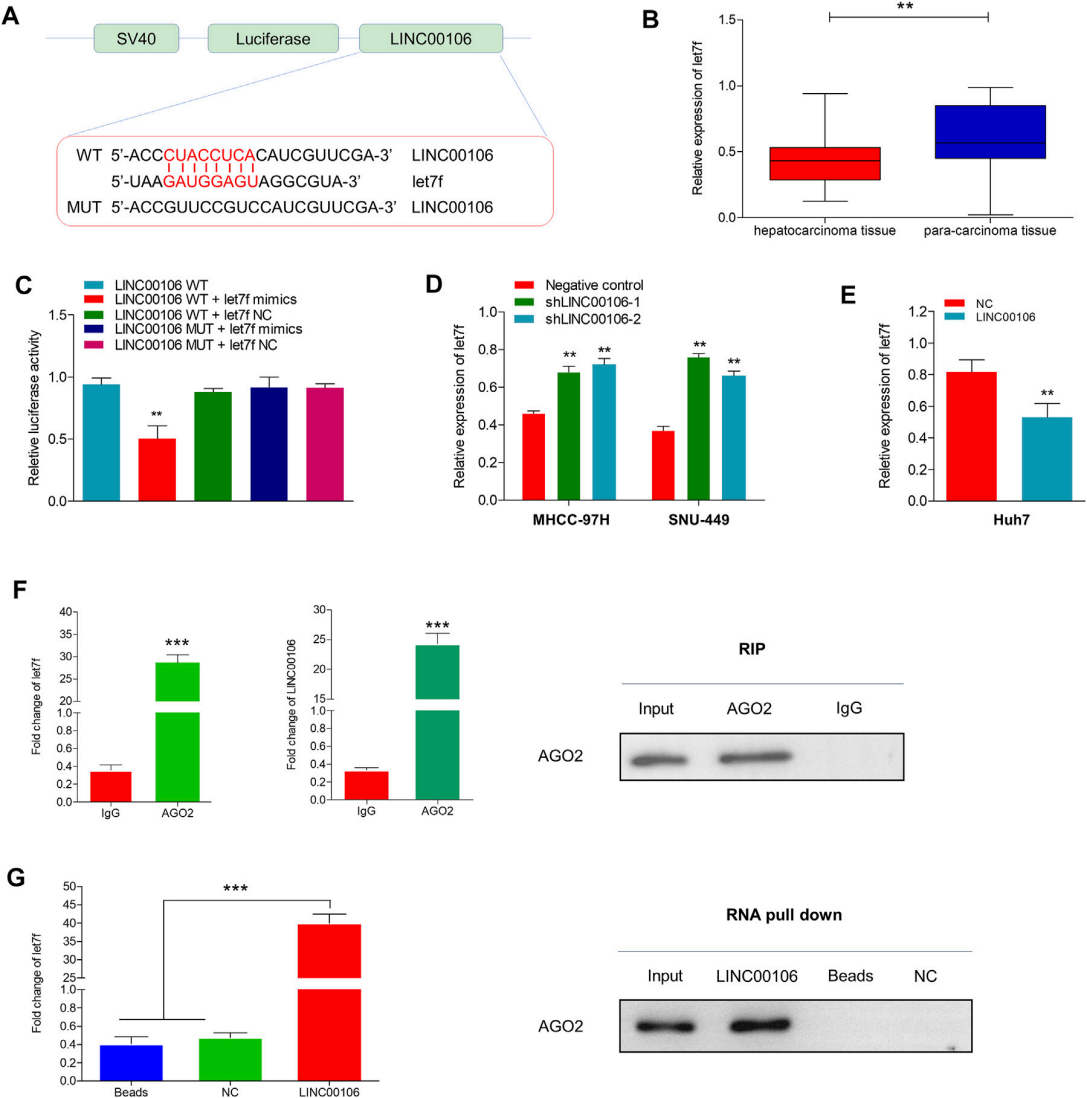
LINC00106 sponges and downregulates let7f in HCC cells. **(A)**, Conjectured binding sites between LINC00106 and let7f by LncBase. **(B)**, Comparison of let7f expression level between 171 pairs of HCC tissues and adjacent normal liver tissues. **(C)**, The relative luciferase activities in 293T cells following the indicated transfection. **(D,E)**, Expression of let7f after LINC00106 manipulation. **(F)**, RIP assay for the relative enrichment of LINC00106 and let7f in anti-IgG or anti-AGO2 specific immuneprecipitates. **(G)**, RNA pull-down assay was used to detect the interaction between LINC00106, let7f and AGO2 (**p < 0.01, ***p < 0.001).

lncRNAs can serve as molecular sponges for microRNAs (miRNAs), which can thus prevent miRNA-mediated suppression of downstream target gene expression through the competitive endogenous RNA (ceRNA) mechanism. By using the bioinformatics database LncBase Predicted v.2, we found that LINC00106 and miR-let7f (let7f) sequences may have the potential binding relationship (Figure 4A). Additionally, we found that let7f was significantly and lowly expressed in HCC tissues (Figure 4B). The dual luciferase reporter gene assay indicated that the relative luciferase activity was weakened in the wild-type LINC00106–let7f mimic-co-transfected HCC cells, whereas it was normal in the mutant type (MUT) LINC00106–let7f mimic-co-transfected cells (Figure 4C). Additionally, the let7f level was markedly elevated in HCC cells with LINC00106 knockdown (MHCC-97H and SNU-449) (Figure 4D), whereas in Huh7 cells overexpressing LINC00106, a decline in the let7f level was observed (Figure 4E). Thus, presumably, LINC00106 negatively regulated let7f and further inhibited RISC-mediated downstream mRNA silencing. We further performed RIP and RNA pull-down assays to confirm this hypothesis. The RIP assay results (Figure 4F) indicated a significantly higher degree of enrichment of LINC00106 and let7f in the Ago2 precipitate than in the IgG control group. The foregoing finding was further verified through a subsequent RNA pull-down assay (Figure 4G). Overall, these data indicated that supported by the ceRNA mechanism, LINC00106 could act as a sponging molecule for let7f.

### Periostin is a Target of Let7f, Which can Mediate the LINC00106-Induced Stemness and Metastasis Properties in HCC Cells

**FIGURE 5:**
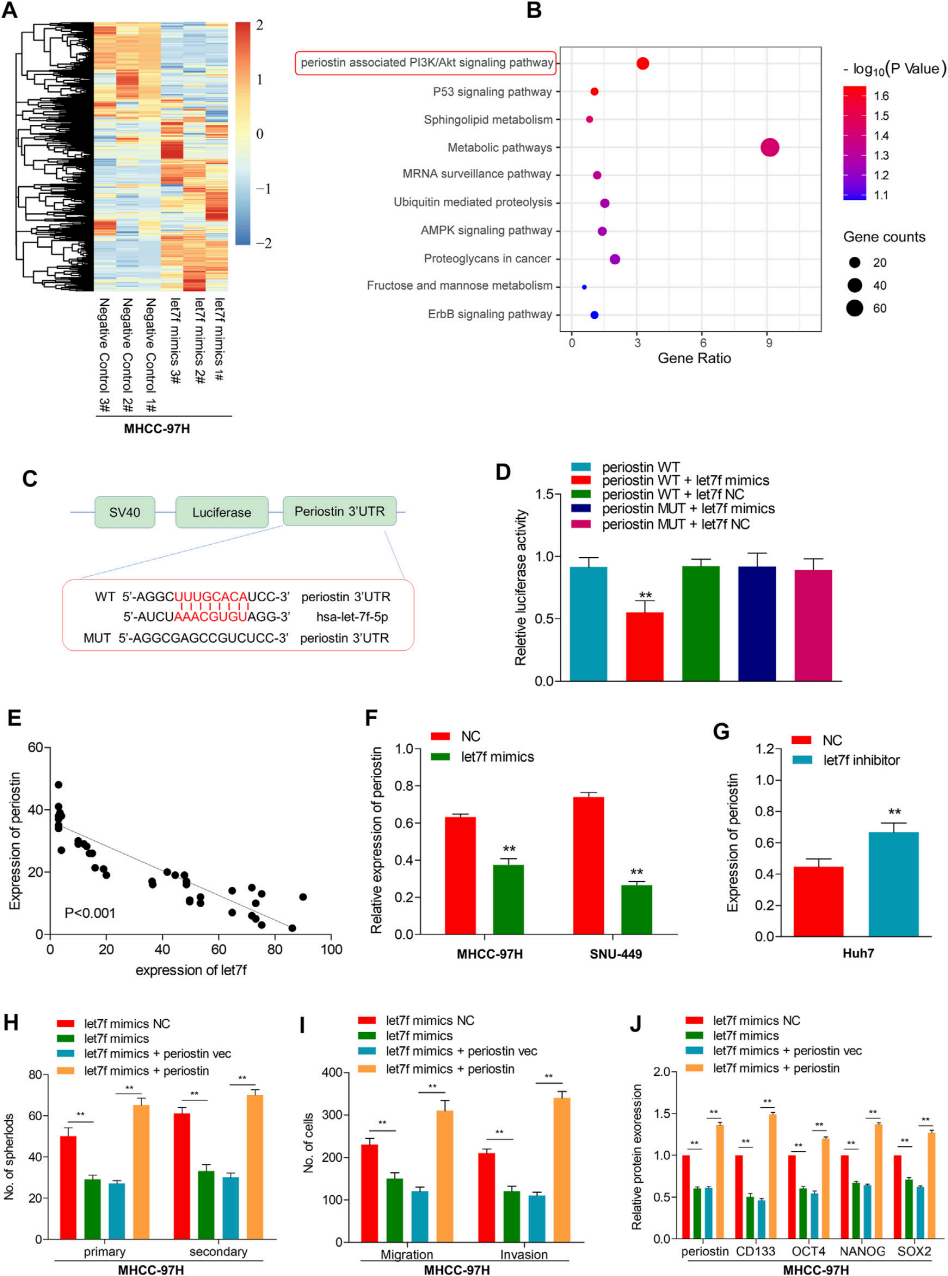
let7f targets to periostin in HCC to regulate the LINC00106-induced stemness and metastasis properties in HCC cells. **(A)**, Heatmap summarizing the differentially expressed genes in let7f overexpressed MHCC-97H cells. **(B)**, KEGG analysis for enriched pathways in let7f overexpressed MHCC-97h cells. **(C)**, The potential binding between let7f and periostin sequences predicted by TargetScan. **(D)**, The relative luciferase activities in 293T cells co-transfected with let7f mimics or miR-NC and luciferase reporter vectors periostin-WT or periostin-Mut. **(E)**, Regression curve with respect to expression of let7f and periostin. **(F,G)**, Expression of periostin protein after knockdown or overexpression of let7f. **(H–J)**. Sphere formation capacities **(H)**, invasion and migration assays **(I)** and expression of stemness biomarkers **(J)** in MHCC-97H cells co-transfected with let7f mimics or let7f mimics NC and periostin or periostin-vec (**p < 0.01).

To determine the role of the LINC00106/let7f axis in HCC, a RNA-seq analysis was performed to profile the gene expressions of let7f mimic–transfected MHCC-97H cells and negative control (Figure 5A). Pathway analysis based on the Kyoto Encyclopedia of Genes and Genomes (KEGG) indicated that the periostin-associated PI3K-AKT axis was most prominently enriched following transfection with let7f mimics (Figure 5B). Meanwhile, based on aforementioned results, we found a markedly decreased periostin level following let7f transfection. Additionally, the analysis using TargetScanHuman 7.2 indicated that let7f and periostin sequences share a potential binding relationship (Figure 5C). The dual luciferase reporter gene assay demonstrated that the relative luciferase activity was weakened in the wild-type periostin–let7f mimic-co-transfected HCC cells, whereas the activity was normal in the MUT periostin–let7f mimic-co-transfected cells (Figure 5D). The mRNA levels of periostin and let7f were found to be negatively correlated (Figure 5E). Moreover, transfection with the let7f mimic was found to lower the expression of periostin at protein levels (Figure 5F), however, transfection with the let7f inhibitor provided an opposite result (Figure 5G). Also, we found that periostin was high expressed in HCC tissues, which is the same as Ki67 (Supplementary Figures S2G,H).

To verify the role of periostin in the regulation of stemness and metastasis properties through let7f in HCC cells, we restored periostin expression in let7f mimics-transfected cells. Periostin overexpression in HCC cells could alleviate the inhibitory effects of let7f mimic on stemness markers (CD133, OCT4, NANOG, and SOX-2) (Figure 5H). Additionally, periostin overexpression could blunt the generation of spheres induced by let7f mimics and suppression of metastatic properties (Figures 5I,J). These data indicated that periostin is a target of let7f, which can mediate the LINC00106-induced stemness and metastasis properties in HCC cells.

### Let7f/Periostin/PI3K Signaling Pathway Mediates LINC00106-Induced HCC Stemness and Metastasis Properties

**FIGURE 6:**
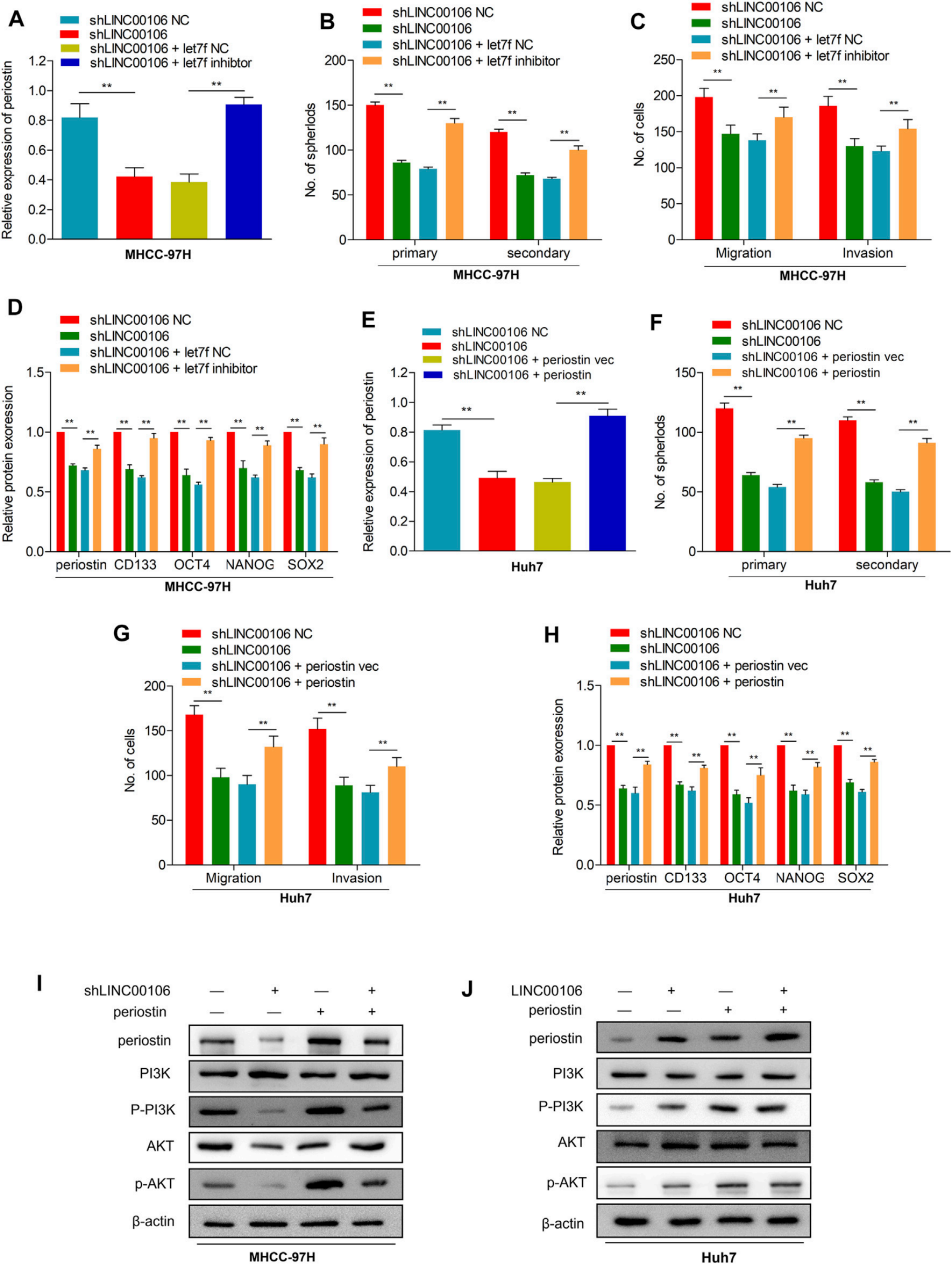
let7f/Periostin/PI3K signaling pathway mediates LINC00106 promoted HCC stemness and metastasis properties. **(A–D)**, Expression of periostin **(A)**, Sphere formation capacities **(B)**, invasion and migration assays **(C)** and expression of stemness biomarkers **(D)** in MHCC-97H cells co-transfected with let7f inhibitors or let7f inhibitors NC and sh-LINC00106 or sh-LINC00106 NC; **(E–H)**. Expression of periostin **(E)**, Sphere formation capacities **(F)**, invasion and migration assays **(G)** and expression of stemness biomarkers **(H)** in Huh7 cells co-transfected with periostin NC or periostin-vec and sh-LINC00106 or sh-LINC00106 NC; **(I,J)**. Expression of periostin, total and phosphorylated PI3K/AKT3 following co-transfected with periostin-vec and sh-LINC00106 or LINC00106 mimics in HCC cell lines (**p < 0.01).

To explore the potential role of let7f/periostin/PI3K pathway in HCC carcinogenesis regulated by LINC00106, we further co-transfected the cells with sh-LINC00106 and let7f inhibitor or induced periostin overexpression. Co-transfection with sh-LINC00106 and let7f inhibitor or periostin overexpression could significantly reverse the inhibition of periostin expression (Figures 6A,E; Supplementary Figures S1A,E) to reverse the LINC00106 knockdown-induced decline in stemness-related markers, such as CD133, NANOG, SOX-2, and OCT4 (Figures 6D,H; Supplementary Figures S1D,H). The sphere formation and invasion and migration assays further verified the role of periostin overexpression or let7f inhibitor in reversing the LINC00106 knockdown-induced decline in stemness and metastatic properties (Figures 6B,C,F,G; Supplementary Figures S1B,C,F,G). Furthermore, the AKT and PI3K phosphorylation was remarkably inhibited by the knockdown of LINC00106 (Figure 6I), whereas it was enhanced by LINC00106 overexpression (Figure 6J). The foregoing findings implied that the let7f/periostin/PI3K axis mediates LINC00106-induced stemness and metastasis properties in HCC cells.

### Mettl3-Mediated M^6^A Modification Participates in LINC00106 Upregulation

**FIGURE 7:**
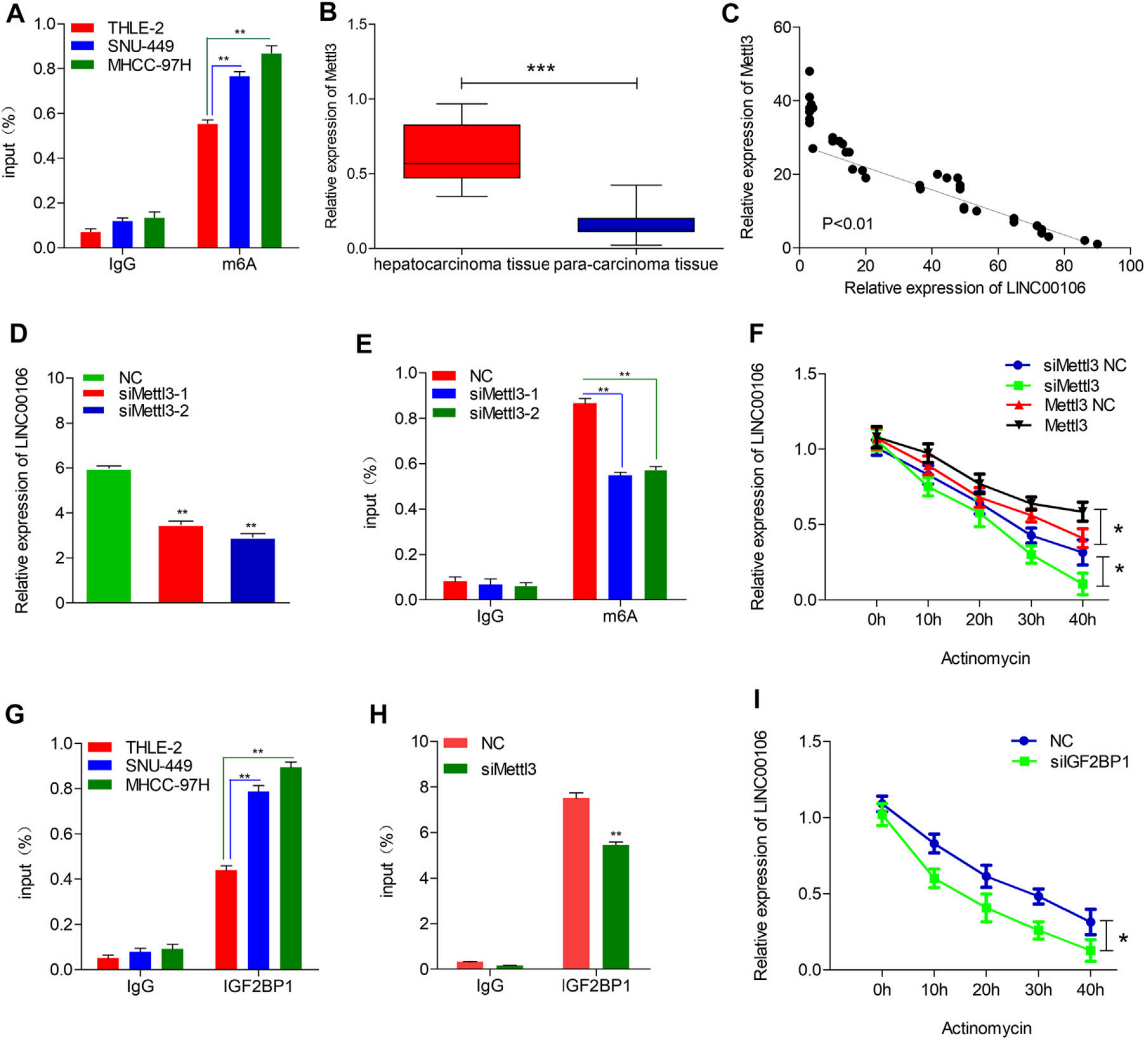
Mettl3-induced m^6^A modification is involved in the upregulation of LINC00106. **(A)**, RIP-qPCR showing the stronger enrichment of m^6^A in SNU-449 and MHCC-97H cells than that in THLE-2 cell. **(B)**, Mettl3 expression was assessed in the HCC tissues and adjacent normal liver tissues. **(C)**, LINC00106 expression was positively correlated with Mettl3 expression in an analysis of HCC tissues. **(D)**, qRT-PCR of LINC00106 after Mettl3 inhibition in SNU-449 cells. **(E)**, RIP-qPCR showing the enrichment of m^6^A in SNU-449 after Mettl3 depletion. **(F)**, The expression of LINC00106 after treatment with 2.5 μM actinomycin D for indicated times, with depletion or overexpression of Mettl3 in SNU-449 cells. **(G)**, RIP-qPCR showing the stronger enrichment of IGF2BP1 in SNU-449 and MHCC-97H cells than that in THLE-2 cell. **(H)**, RIP-qPCR showing the enrichment of IGF2BP1 in SNU-449 with Mettl3 knockdown. **(I)**, The expression of LINC00106 after treatment with 2.5 μM actinomycin D for indicated times, with IGF2BP1 knockdown in SNU-449 cells (*p < 0.05, **p < 0.01).

To explore the mechanism leading to ectopic expression of LINC00106, we used RMVar (http://rmvar.renlab.org) prediction to reveal that numerous m^6^A sites were distributed in LINC00106 with high confidence. Hence, we further clarified whether m^6^A modification could upregulate the expression of LINC00106. By using the m^6^A RNA IP (RIP) assay along with qRT-PCR, we found that m^6^A was more significantly enriched in MHCC-97H and SNU-449 cells than in normal THLE-2 cell (Figure 7A). Methyltransferase like 3 (Mettl3) is a well-known m^6^A methyltransferase (“writer”) in mammalian cells. To verify the results, the expression of Mettl3 and its association with LINC00106 were assessed based on the TCGA database and HCC tissues expression detection. The results indicated that the Mettl3 level in HCC tissues was prominently elevated (Figure 7B), and Mettl3 and LINC00106 expressions were positively correlated (R = 0.53, *p* < 0.01; Figure 7C). Moreover, silencing of Mettl3 lead to a significant reduction in LINC00106 expression (Figure 7D). RIP assays revealed that m^6^A modification of LINC00106 expression was reduced upon Mettl3 silencing (Figure 7E). Furthermore, Mettl3 could regulate the stability of LINC00106 in HCC cells. Following the blockage of RNA *de novo* synthesis by actinomycin D treatment of cells, the stability of LINC00106 was decreased with the depletion of Mettl3. By contrast, the stability of LINC00106 was increased with the overexpression of Mettl3 (Figure 7F). The findings revealed that in HCC cells, Mettl3 could specifically maintain the stability of LINC00106 by facilitating m^6^A modification.

**FIGURE 8:**
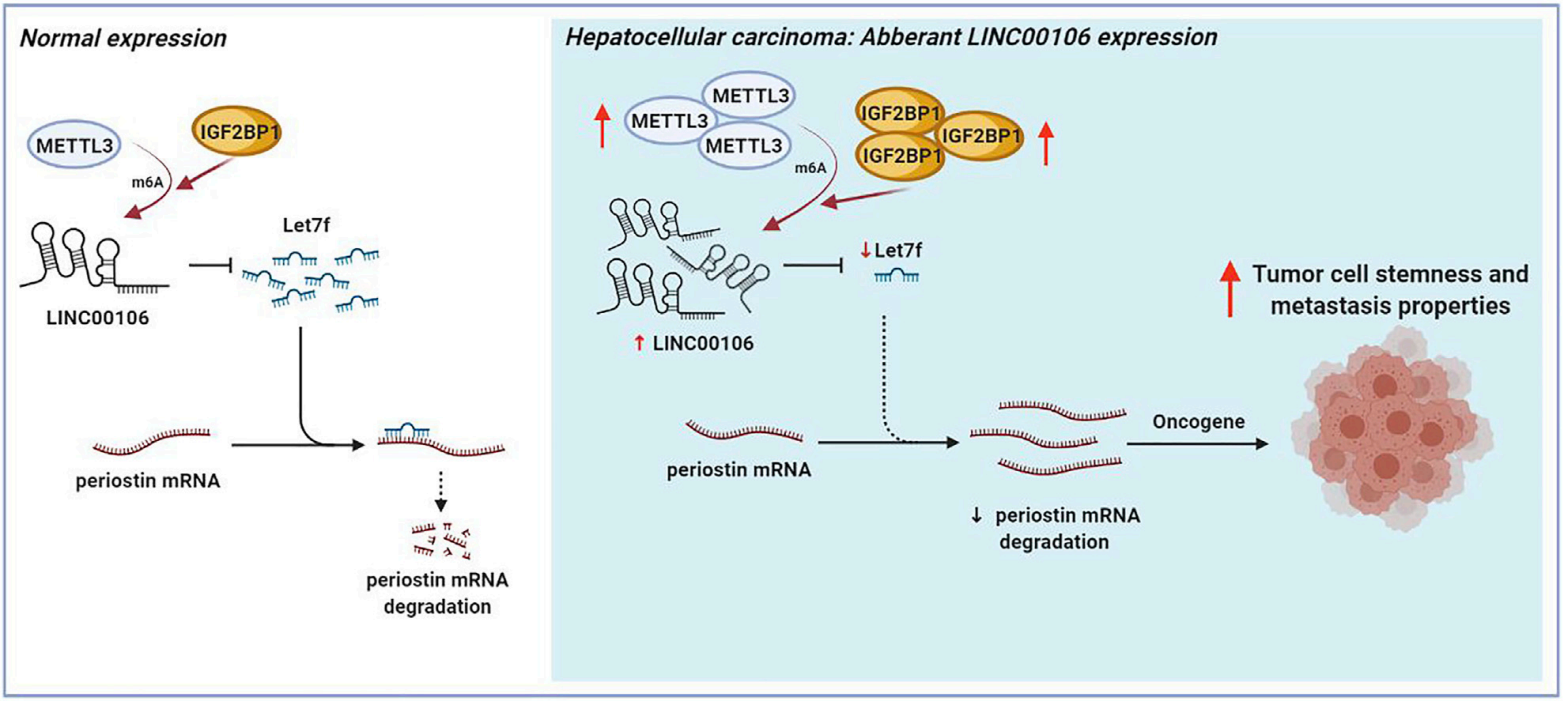
The schematic figure shows that m^6^A-mediated upregulation of LINC00106 promotes hepatocellular carcinoma stemness and metastasis properties via sponging let7f.

The mechanism underlying the Mettl3-mediated LINC00106 stability in HCC cells was explored in detail in this study. The specific function of the insulin-like growth factor 2 mRNA-binding protein 1 (IGF2BP1; an m^6^A “reader”) in governing the stability of m^6^A-modified lncRNAs in carcinomas has already been reported (Jonas et al., 2020). Using RMVar, the probable readers of LINC00106 m^6^A loci were estimated, in which the binding sites of IGF2BP1 on LINC00106 with high confidence were found. RIP and qRT-PCR assays confirmed a direct interaction between IGF2BP1 and LINC00106 in HCC cells (Figure 7G). Additionally, upon inhibiting Mettl3, the IGF2BP1–LINC00106 interplay was disrupted (Figure 7H). We further used actinomycin D to treat cells and discovered a prominently lowered LINC00106 level following depletion of IGF2BP1 (Figure 7I). The results suggested that IGF2BP1 made LINC00106 more stable by binding to it, thereby displaying m^6^A dependence. Figure 8 illustrates the mechanism concerning the regulation of stemness and metastatic properties in HCC cells by m^6^A-induced LINC00106.

## Discussion

Recurrence and metastasis in HCC caused by the stemness and metastasis properties of HCC cells not only seriously affects the survival and prognosis of patients but also enhances the requirements and challenges for diagnosis and treatment programmes (Kelley and Greten, 2021). Moreover, the underlying mechanism through which HCC cells acquire stemness and metastasis properties remains poorly understood to date.

Numerous studies have proved that the gain and loss of function of selected lncRNAs can affect the cellular processes, development, and diseases (Atianand et al., 2016; Andersen et al., 2019). One of the greatest discoveries during the postgenomic age has been the discovery of a broad, novel spectrum of human genomic regulators known as lncRNAs, whose expression patterns are exquisitely specific to the cell type in healthy and diseased states (Huo et al., 2017; Sarropoulos et al., 2019; Rinn and Chang, 2020). Several lncRNAs from human HCCs, which have been demonstrated to facilitate or suppress metastasis in carcinomas by using sequestering EMT-associated miRNAs as decoying molecules (Wong et al., 2018) or using self-renewal simulation of hepatoma stem cells (Wang et al., 2015; Wu et al., 2019). To date, none of the studies have reported the expression of LINC00106 in HCC. In the present study, LINC00106 was found to be remarkably upregulated in HCC, and patients with a high LINC00106 level displayed a poor prognosis. When the LINC00106 levels were decreased, the stemness and metastasis properties of HCC cells were suppressed *in vitro* and *in vivo*, and the stemness and EMT markers were significantly downregulated in HCC cells. Our results suggest that LINC00106 can play an oncogenic role and confer the stemness and metastasis properties to HCC cells.

Based on the aforementioned findings, we further explored the underlying molecular mechanism by which LINC00106 modulated the stemness and metastasis properties in HCC cells. Numerous studies have shown that the molecular sponges acting as miRNAs are the main regulators of lncRNAs (He et al., 2020; Statello et al., 2020). Considering the abundances of LNC00106 in the nucleus and cytoplasm, we speculate that the ceRNA mechanism may also be involved in the carcinogenic effect of LNC00106 in HCC. In order to confirm the above hypothesis, purported let7f binding sites for LINC00106 were revealed through the bioinformatics analysis, and the luciferase reporter, RIP, and RNA pull-down assays were further performed. Our results inferred that LINC00106 is capable of sponging and declining the expression of let7f through ceRNA mechanism in HCC. Additionally, the LINC00106/let7f axis could further regulate the periostin mRNA level. Periostin, as a stromal factor for healthy stem cell micro-environment, plays a crucial role during metastatic colonization for the maintenance and expansion regulation of CSCs to promote tumor metastasis (Wang and Ouyang, 2012; Zhou et al., 2015). We found that periostin overexpression in HCC cells could increase the expression of stemness markers, blunt let7f mimic-triggered sphere formation, and metastasis properties inhibition to promote HCC stemness and metastasis potential. These biological characteristics are regulated by the periostin-associated PI3K-AKT signaling pathway.

As an emerging layer of epigenetic regulation, the RNA m^6^A can influence the fate of modified RNA molecules, which exert crucial effects on nearly all the vital bioprocesses, including cancer development (Huang et al., 2020). One critical member of the m^6^A complex is Mettl3, which is an m^6^A “writer”. Studies have reported that METTL3 can repress SOCS2 expression in HCC through an m^6^A-YTHDF2-dependent mechanism and contributes to HCC progression (Chen et al., 2018). In the present study, Mettl3 was found to specifically maintain LINC00106 stability by facilitating m^6^A modification in HCC cells, so as to increase the abundances of LINC00106 in the nucleus, which can furtherly promote stemness and metastasis properties in HCC. The biological functions of the m^6^A modification are mediated through the binding of m^6^A to IGF2BP1 and other m^6^A “readers”. An m^6^A-dependent stimulation of SRF expression by IGF2BP1 has been reported, which was accomplished through disruption of SRF mRNA decay directed by miRNAs, consequently resulting in improved action of SRF-dependent transcription and carcinoma cell proliferation and infiltration (Müller et al., 2018). Our findings revealed that IGF2BP1 could directly bind to LINC00106 and promote LINC00106 expression in an m^6^A-dependent manner.

In summary, we demonstrated that LINC00106 can play an oncogenic role and promote stemness and metastasis in HCC. Moreover, LINC00106 can sponge and reduce the expression of miR-let7f in HCC through ceRNA mechanism to regulate the periostin-associated PI3K-AKT signaling pathway. Additionally, the m^6^A modification mediated by Mettl3 resulted in abnormal LINC00106 overexpression. Our findings not only demonstrate the mechanism by which LINC00106 regulates stemness but also offer potential therapeutic targets to deal with tumor metastasis in HCC.

## Conclusion

We demonstrated that LINC00106 can play an oncogenic role and promote stemness and metastasis properties in HCC. Mechanistically, through ceRNA mechanism, LINC00106 is capable of sponging and declining the expression of miR–let7f in HCC, so that under both *in vivo* and *in vitro* conditions, the stemness and metastatic properties of HCC cells can be augmented by the periostin-associated PI3K-AKT signaling pathway. Moreover, aberrant LINC00106 overexpression was induced by Mettl3-mediated m^6^A modification. Thus, the findings of this study suggest that LINC00106 is a potential diagnostic marker and therapeutic target for HCC.

## Data Availability

The datasets presented in this study can be found in online repositories. The names of the repository/repositories and accession number(s) can be found in the article/Supplementary Material.

## References

[B1] Andersen R. E. Hong S. J. Lim J. J. Cui M. Harpur B. A. Hwang E. (2019). The Long Noncoding RNA Pnky Is a Trans-acting Regulator of Cortical Development *In Vivo* . Dev. Cel 49 (4), 632–642. 10.1016/j.devcel.2019.04.032 PMC6556063 31112699

[B2] Atianand M. K. Hu W. Satpathy A. T. Shen Y. Ricci E. P. Alvarez-Dominguez J. R. (2016). A Long Noncoding RNA lincRNA-EPS Acts as a Transcriptional Brake to Restrain Inflammation. Cell 165 (7), 1672–1685. 10.1016/j.cell.2016.05.075 27315481 PMC5289747

[B3] Chen G. Wang Y. Zhao X. Xie X.-z. Zhao J.-g. Deng T. (2021). A Positive Feedback Loop between Periostin and TGFβ1 Induces and Maintains the Stemness of Hepatocellular Carcinoma Cells via AP-2α Activation. J. Exp. Clin. Cancer Res. 40 (1). 10.1186/s13046-021-02011-8 PMC8243733 34193219

[B4] Chen M. Wei L. Law C. Tsang F. H. Shen J. Cheng C. L. (2018). RNA N6-Methyladenosine Methyltransferase-like 3 Promotes Liver Cancer Progression through YTHDF2-dependent Posttranscriptional Silencing of SOCS2. Hepatology 67 (6), 2254–2270. 10.1002/hep.29683 29171881

[B5] Faltas B. (2012). Cornering Metastases: Therapeutic Targeting of Circulating Tumor Cells and Stem Cells. Front. Oncol. 2. 10.3389/fonc.2012.00068 PMC3388423 22783544

[B6] He H. Wang Y. Ye P. Yi D. Cheng Y. Tang H. (2020). Long Noncoding RNA ZFPM2-AS1 Acts as a miRNA Sponge and Promotes Cell Invasion through Regulation of miR-139/GDF10 in Hepatocellular Carcinoma. J. Exp. Clin. Cancer Res. 39 (1). 10.1186/s13046-020-01664-1 PMC7427719 32795316

[B7] Huang H. Weng H. Chen J. (2020). M6A Modification in Coding and Non-coding RNAs: Roles and Therapeutic Implications in Cancer. Cancer Cell 37 (3), 270–288. 10.1016/j.ccell.2020.02.004 32183948 PMC7141420

[B8] Huo X. Han S. Wu G. Latchoumanin O. Zhou G. Hebbard L. (2017). Dysregulated Long Noncoding RNAs (lncRNAs) in Hepatocellular Carcinoma: Implications for Tumorigenesis, Disease Progression, and Liver Cancer Stem Cells. Mol. Cancer 16 (1). 10.1186/s12943-017-0734-4 PMC5651571 29061150

[B9] Jonas K. Calin G. A. Pichler M. (2020). RNA-binding Proteins as Important Regulators of Long Non-coding RNAs in Cancer. Ijms 21 (8), 2969. 10.3390/ijms21082969 PMC7215867 32340118

[B10] Kelley R. K. Greten T. F. (2021). Hepatocellular Carcinoma - Origins and Outcomes. N. Engl. J. Med. 385 (3), 280–282. 10.1056/nejmcibr2106594 34260842

[B11] Ling S. Shan Q. Zhan Q. Ye Q. Liu P. Xu S. (2019). USP22 Promotes Hypoxia-Induced Hepatocellular Carcinoma Stemness by a HIF1α/USP22 Positive Feedback Loop upon TP53 Inactivation. Gut 69 (7), 1322–1334. 10.1136/gutjnl-2019-319616 31776228

[B12] Ma X.-L. Hu B. Tang W.-G. Xie S.-H. Ren N. Guo L. (2020). CD73 Sustained Cancer-Stem-Cell Traits by Promoting SOX9 Expression and Stability in Hepatocellular Carcinoma. J. Hematol. Oncol. 13 (1). 10.1186/s13045-020-0845-z PMC7003355 32024555

[B13] Müller S. Glaß M. Singh A. K. Haase J. Bley N. Fuchs T. (2018). IGF2BP1 Promotes SRF-dependent Transcription in Cancer in a m6A- and miRNA-dependent Manner. Nucleic Acids Res. 47 (1), 375–390. 10.1093/nar/gky1012 PMC6326824 30371874

[B14] Rinn J. L. Chang H. Y. (2020). Long Noncoding RNAs: Molecular Modalities to Organismal Functions. Annu. Rev. Biochem. 89 (1), 283–308. 10.1146/annurev-biochem-062917-012708 32569523

[B15] Sarropoulos I. Marin R. Cardoso-Moreira M. Kaessmann H. (2019). Developmental Dynamics of lncRNAs across Mammalian Organs and Species. Nature 571 (7766), 510–514. 10.1038/s41586-019-1341-x 31243368 PMC6660317

[B16] Shu G. Su H. Wang Z. Lai S. Wang Y. Liu X. (2021). LINC00680 Enhances Hepatocellular Carcinoma Stemness Behavior and Chemoresistance by Sponging miR-568 to Upregulate AKT3. J. Exp. Clin. Cancer Res. 40 (1). 10.1186/s13046-021-01854-5 PMC7836199 33499874

[B17] Statello L. Guo C.-J. Chen L.-L. Huarte M. (2020). Gene Regulation by Long Non-coding RNAs and its Biological Functions. Nat. Rev. Mol. Cel Biol 22 (2), 96–118. 10.1038/s41580-020-00315-9 PMC7754182 33353982

[B18] Wang Y. He L. Du Y. Zhu P. Huang G. Luo J. (2015). The Long Noncoding RNA lncTCF7 Promotes Self-Renewal of Human Liver Cancer Stem Cells through Activation of Wnt Signaling. Cell Stem Cell 16 (4), 413–425. 10.1016/j.stem.2015.03.003 25842979

[B19] Wang Z. Ouyang G. (2012). Periostin: A Bridge between Cancer Stem Cells and Their Metastatic Niche. Cell Stem Cell 10 (2), 111–112. 10.1016/j.stem.2012.01.002 22305559

[B20] Wong C.-M. Tsang F. H.-C. Ng I. O.-L. (2018). Non-coding RNAs in Hepatocellular Carcinoma: Molecular Functions and Pathological Implications. Nat. Rev. Gastroenterol. Hepatol. 15 (3), 137–151. 10.1038/nrgastro.2017.169 29317776

[B21] Wu J. Zhu P. Lu T. Du Y. Wang Y. He L. (2019). The Long Non-coding RNA LncHDAC2 Drives the Self-Renewal of Liver Cancer Stem Cells via Activation of Hedgehog Signaling. J. Hepatol. 70 (5), 918–929. 10.1016/j.jhep.2018.12.015 30582981

[B22] Yuan J.-h. Liu X.-n. Wang T.-t. Pan W. Tao Q.-f. Zhou W.-p. (2017). The MBNL3 Splicing Factor Promotes Hepatocellular Carcinoma by Increasing PXN Expression through the Alternative Splicing of lncRNA-PXN-AS1. Nat. Cel Biol 19 (7), 820–832. 10.1038/ncb3538 28553938

[B23] Zheng A. Song X. Zhang L. Zhao L. Mao X. Wei M. (2019). Long Non-coding RNA LUCAT1/miR-5582-3p/TCF7L2 axis Regulates Breast Cancer Stemness via Wnt/β-Catenin Pathway. J. Exp. Clin. Cancer Res. 38 (1). 10.1186/s13046-019-1315-8 PMC6626338 31300015

[B24] Zhou W. Ke S. Q. Huang Z. Flavahan W. Fang X. Paul J. (2015). Periostin Secreted by Glioblastoma Stem Cells Recruits M2 Tumour-Associated Macrophages and Promotes Malignant Growth. Nat. Cel Biol. 17 (2), 170–182. 10.1038/ncb3090 PMC4312504 25580734

[B25] Zuo X. Chen Z. Gao W. Zhang Y. Wang J. Wang J. (2020). M6A-mediated Upregulation of LINC00958 Increases Lipogenesis and Acts as a Nanotherapeutic Target in Hepatocellular Carcinoma. J. Hematol. Oncol. 13 (1). 10.1186/s13045-019-0839-x PMC6951025 31915027

