# Supplementary material for: M6A-Mediated Upregulation of LINC00106 Promotes Stemness and Metastasis Properties of Hepatocellular Carcinoma via Sponging Let7f
Source: Front Cell Dev Biol. 2021 Nov 11;9:781867. doi: 10.3389/fcell.2021.781867 (PMC8632228; doi:10.3389/fcell.2021.781867)
Supplement: Supplementary file 1 [file DataSheet1.doc]

Annexed file 2

**Immunohistochemistry (IHC)**

The surgically resected paraffin specimens were collected from the Department of Pathology of the Affiliated Hospital of Guilin Medical University and were prepared into 4‑mm thick slices. According to the EnVision two‑step procedure, in Briefly, dewaxing and hydration were carried out. Subsequently a primary antibody against Periostin (Abcam) with a dilution of 1:100 was added, and the specimens were incubated at room temperature for 1 h, then washed and incubated at room temperature for 0.5 h with a secondary antibody (MXB Biotechnologies, Fuzhou, China). A DAB Horseradish Peroxidase Color Development kit (Beyotime Institute of Biotechnology, Haimen, China) was used for color development and scored using the Olympus X71 inverted microscope (Olympus Corp., Tokyo, Japan). Based on the staining intensity, samples were divided into the following grades: 0, <10% of tumor cells were positively stained; 1+, 11‑25% of tumor cells were positively stained; 2+, 26‑50% of tumor cells were positively stained; and 3+, >50% of tumor cells were positively stained. Immunohistochemical analysis and scoring were conducted by two researchers, respectively.

**Western blot analysis**

Cell samples were collected and lysis buffer [50 mM Tris‑HCl, 137 mM NaCl, 10% glycerol, 100 mM sodium orthovanadate, 1 mM phenylmethylsulfonyl fluoride (PMSF), 10 mg/ml aprotinin, 10 mg/ml leupeptin, 1% Nonidet P‑40, and 5 mM protease inhibitor cocktail; pH 7.4] was added to extract total proteins. Then the protein concentration was assessed by BCA (bicinchoninic acid) kit (Beyotime Institute of Biotechnology) and the appropriate amount of loading buffer was added. Protein sample (30 µg) was added to 10% sodium dodecyl sulfate-polyacrylamide gel electrophoresis system (SDS-PAGE) for electrophoresis separation and then transferred to polyvinylidene difluoride (PVDF) membranes (Bio‑Rad Laboratories, Inc., Hercules, CA, USA). Then, 5% non‑fat milk was used to incubate the PVDF membranes, and peroxidase-labeled secondary antibodies (HRP-labeled goat anti‑rabbit IgG; cat. no. A0208; Beyotime Institute of Biotechnolgy) at a 1:10,000 were used for incubation for 1h at room temperature. Subsequently, chemiluminescence was used to display the imprinting, and the results were analyzed using the Tannon 5200 chemiluminescent imaging system (Tanon Science and Technology, Shanghai, China).

**Dual luciferase reporter assay**

The sequences of wild- or mutant-type periostin 3′-UTR were inserted into PmiRGLO dural-luciferase reporters. Thereafter, the recombinant plasmids were co-transfected with miR-let7f mimics or miRNA mimics negative control (NC) into 293 T cells by lipofectamine TM 3000 (Thermo Fisher). After 36 h transfection, Luciferase assay system (Promega, Madison, USA) was used to determine the relative luciferase activity normalizing to renilla luciferase activity. The binding between LINC00106 and miR-let7f was verified using a similar method.

**Quantitative real time polymerase chain reaction (qRT-PCR)**

The collected cells and tissues were added to TRIzol (Invitrogen; Thermo Fisher Scientific, Inc.) and total RNA was extracted according to the RNAiso Plus kit instructions (Takara Bio, Inc., Otsu, Japan). The RNA concentration was assessed using the NanoDrop spectrophotometer (Thermo Fisher Scientific, Inc.). The FastQuant cDNA First‑Strand Synthesis kit (Tiangen Biotech, Co., Ltd., Beijing, China) was used for reverse transcription. A qRT‑PCR kit FastStart Universal SYBR Green Master Mix (Rox) (Roche Diagnostics GmbH, Mannheim, Germany) was used to produce the reaction system and amplification according to the manufacturer's instructions. The reaction cycling conditions were performed as follows: 1 cycle at 95˚C for 15 min, followed by 40 cycles at 95˚C for 10 sec and at 60˚C for 60 sec, and then relative mRNA expression was analyzed using the comparative quantification cycle method followed by normalization to β-actin expression (Applied Biosystems; Thermo Fisher Scientific, Inc.). The primers used for amplification were as follows in Annexed file 1.

**Plate cloning experiment**

The prepared cell suspension was seeded on a 6-well plate at 600 cells/well. Cell colonies were formed after being cultured for 1~2 weeks. Cells were fixed with 4% paraformaldehyde for 20 min and stained overnight with 1% crystal violet (cat. no. G1062; Solarbio, Tokyo, Japan). After three washes in PBS, the images of the results were observed and captured using a fluorescence microscope (IX71; Olympus Corporation) and the number of colonies/well was calculated using ImageJ (National Institutes of Health, Bethesda, MD, USA).

**Experiments of cell invasion and migration**

A transwell chamber (8 µm; BD Biosciences) (the chamber for invasion detection was coated with Matrigel, and the chamber for migration detection was without Matrigel) was placed into a 24-well plate with ~600 µl medium containing 10% FBS which was added to the bottom chamber. The prepared cell suspension was seeded in a transwell chamber at 1,000 cells/well. One or two days later, the cell preparation in the small chamber was observed under a fluorescence microscope (IX71; Olympus Corporation). After an appropriate number of cells had passed through the cell pores, the cells were fixed with 4% paraformaldehyde and stained with 1% crystal violet (cat. no. G1062; Solarbio) and images were captured.

***In vivo* tumour growth assay and nude murine xenograft model**

Forty BALB/c 8-week-old male nude mice weighing 18 g-20 g (20 were used for tumor growth while the other 20 were used for a tumor metastasis model) were purchased from the Animal Experimental Center of Guangxi Medical University and all animals were used in accordance with the operating instructions and approved by the Animal Care Committee of Guangxi Medical University, with room temperature atmosphere and ad libitum. Tumor cell lines in logarithmic growth phase were collected and washed with PBS to prepare a cell suspension. The prepared cell suspension (2x10 ^6^ cells/ml) was subcutaneously injected into the right groin area of 8‑week‑old nude mice. The tumor volume was assessed weekly. Nude mice were sacrificed by cervical dislocation. Twenty‑eight days after surgery, the tumors were excised and the tumor weights were recorded. For the tumor metastasis model, the cells were collected and washed with PBS and then prepared into a cell suspension. The cell suspension with a concentration of 1x10^7^ cells/ml was injected into the tail vein of nude mice. All mice were sacrificed by cervical dislocation 60 days after inoculation.

**RNA-seq analysis**

The quality of total RNA was assessed using the Agilent 2100 Bioanalyzer (Agilent Technologies, Inc., Santa Clara, CA, USA) and the NanoDrop ND‑1000 spectrophotometer. RNA expression was then analyzed using the Affymetrix HU U133 plus 2.0 array according to the manufacturer's instructions. The raw data was normalized by the Robust Multiarray Average (RMA) of expression control platform software (Affymetrix; Thermo Fisher Scientific, Inc.). Scatter plot analysis of the gene with significant changes in LINC00106 in the downregulation group and the control group to screen the genes with upregulation or downregulation of expression ≥3 times was performed. Gene Cluster v3.0 software cluster (Stanford University, Stanford, CA, USA) was used for cluster analysis, and Java TreeView v1.1.4r3 software (Alok Saldanha; http://jtreeview.sourceforge.net/) was used for thermal image visualization.
