# Supplementary material for: M6A-Mediated Upregulation of LINC00106 Promotes Stemness and Metastasis Properties of Hepatocellular Carcinoma via Sponging Let7f
Source: Front Cell Dev Biol. 2021 Nov 11;9:781867. doi: 10.3389/fcell.2021.781867 (PMC8632228; doi:10.3389/fcell.2021.781867)
Supplement: Supplementary file 2 [file Table1.DOC]

Annexed file 1: Primers sequences used in this study

| **Gene** | **Primer** | **Sequence** |
| --- | --- | --- |
| U6 | Forward | CTCGCTTCGGCAGCACA |
|  | Backward | AACGCTTCACGAATTTGCGT |
| POSTN | Forward | GCGAGATCATCAAGCCAGCAGAG |
|  | Backward | ATGTCCAGTCTCCAGGTTGTGTCA |
| beta-actin | Forward | GCGGACTATGACTTAGTTGCGTTACA |
|  | Backward | TGCTGTCACCTTCACCGTTCCA |
| shLINC00106-1 | Sequence | GGAAGACTACAGACTTAACCC |
| shLINC00106-2 | Sequence | GGAAGACTAGAAGCTAACCGC |
| let-7f-5p mimics | Sequence | UGAGGUAGUAGAUUGUAUAGUU |
| mimics control | Sequence | AGCUGAUUUCGUCUUG GUA |
| let-7f-5p inhibitor | Sequence | AACUAUACAAU CUACUACCUCA |
| inhibitor control | Sequence | ACGUGACAC GUUCGGAGAATT |
| Mettl3 siRNA-1 | Sequence | GACGAATTATCAATAAACACACT |
| Mettl3 siRNA-2 | Sequence | CGCAAGATTGAGTTATTTGGACG |
| IGF2BP1 siRNA | Sequence | TTGAATAGAAGCAGAAAAACATT |
